# Controlled Photoanode Properties for Large-Area Efficient and Stable Dye-Sensitized Photovoltaic Modules

**DOI:** 10.3390/nano11082125

**Published:** 2021-08-20

**Authors:** Wei-Hao Chiu, Kun-Mu Lee, Vembu Suryanarayanan, Jen-Fu Hsu, Ming-Chung Wu

**Affiliations:** 1 Center of Green Technology, Chang Gung University, Taoyuan 33302, Taiwan; weihao.chiu@gmail.com; 2 Department of Chemical and Materials Engineering, Chang Gung University, Taoyuan 33302, Taiwan; 3 Division of Neonatology, Department of Pediatrics, Chang Gung Memorial Hospital, Linkou 33305, Taiwan; 4 Electroorganic and Materials Electrochemistry Division, CSIR-Central Electrochemical Research Institute, Karaikudi 630003, India; vidhyasur@yahoo.co.in; 5 School of Medicine, College of Medicine, Chang Gung University, Taoyuan 33302, Taiwan

**Keywords:** dye-sensitized, photovoltaic, solar module, photoanode, large

## Abstract

Nowadays, a dye-sensitized solar cell (DSSC) attracts attention to its development widely due to its several advantages, such as simple processes, low costs, and flexibility. In this work, we demonstrate the difference in device structures between small size and large size cells (5 cm × 5 cm, 10 cm × 10 cm and 10 cm × 15 cm). The design of the photoanode and dye-sensitized process plays important roles in affecting the cell efficiency and stability. The effects of the TiO_2_ electrode, using TiCl_4(aq)_ pretreatment and post-treatment processes, are also discussed, whereas, the open-circuit voltage (Voc), short-circuit current density (Jsc), and module efficiency are successfully improved. Furthermore, the effects on module performances by some factors, such as dye solution concentration, dye soaking temperature, and electrolyte injection method are also investigated. We have demonstrated that the output power of a 5 cm × 5 cm DSSC module increases from 86.2 mW to 93.7 mW, and the module efficiency achieves an outstanding performance of 9.79%. Furthermore, enlarging the DSSC modules to two sizes (10 cm × 10 cm and 10 cm × 15 cm) and comparing the performance with different module designs (C-DSSC and S-DSSC) also provides the specific application of polymer sealing and preparing high-efficiency large-area DSSC modules.

## 1. Introduction

In early 1990s, the dye-sensitized solar cell (DSSC) is introduced by O’Regan and Grätzel [1]. Because of its easy manufacturing, low-cost materials and advantages for color selectivity, transparency and flexibility, DSSC becomes a charming photovoltaic device for wide researchers with huge resources. Until now, DSSCs have been intensely researched for more than two decades, where the highest certified efficiency of a lab-size cell reported to date is 13% [2], and the published highest efficiency achieved is even higher: 14.3% [3]. In the past, the researchers focused on the new novel materials such as dye [4], the electron transport electrode [5,6], HTM (hole transport material) [7], electrolytes [8], the counter electrode (CE) [9,10] and the compatibility of each of these. The mechanisms of the charge transfer and energy transfer in a device with a small area are also deeply discussed to save on material waste and for a more convenient measurement. However, the first important issue to shift from academic research to practical product development is the large-area design and manufacturing method of the device. In the enlarging progress, the key factors of the DSSC module, such as high efficiency, high stability and module design technology, are important technical indicators to commercialize this device. In order to transfer results achieved with small laboratory cells to a full production line for dye-sensitized solar modules to be used for practical applications, all the process steps and technological parameters that are relevant to industrial production have to be investigated [11]. Several major advancements have been made to upscale the DSSC module in terms of their various interconnection designs, material components, scalable fabrication processes, outdoor/indoor stability testing, tandem cells/modules to absorb a wider range of light over the solar spectrum, and innovative applications such as a hybrid energy harvesting and storage devices.

With regard to the enlarging of the DSSC module, the module is composed of increasing individual unit cells. The relatively low sheet resistance of the transparent conductive coating (TCO), similar to SnO_2_:F (FTO) (usually lower than 10 Ω/square) and used as the current collector, limits the width of the individual cells to less than 1 cm [9]. One method to reduce the ohmic resistance losses in a module is to interconnect many parallel single cells in series, such as the Z-type [12], W-type [13] and the monolithic connection of individual cells. The goal of pattern design is the balancing between the electron collection efficiency and maximizing the area of the working electrode, the so-called active area, to achieve the maximum power output. In addition to the above designs, a different design such as the S-shape should be proposed. The alternative method is to apply low resistivity metals, like Ag, Cu, Ti, etc., as the electron collector grid on substrates of DSSC modules. In order to increase the collection efficiency of electrons, the length of the electrode and electrons transfer should be optimized, quite similar to silicon technology [14].

The working electrode is the core part of DSSCs, where the dye absorption and the site of electrolyte reduction, as well as the excitation of the dye, take place. The electrode plays an important role in photoelectric conversion, electron transfer and dye redox reactions. Although the surface treatment of titanium dioxide electrodes and its effects has been discussed in many previous studies [15,16], TiCl_4(aq)_ treatment to increase the electron collection ability and dye absorption capability is the most common and effective method. The detailed TiCl_4(aq)_ treatment process is described in the Materials and Methods section [17]. In addition to the TiCl_4_ post-treatment, the plasma reduction reported by Cho [18] shows a good efficiency enhancement attributed by higher electron diffusion coefficients and a lower recombination rate. However, most of the literature is focused on small-area DSSC cells. There are significant differences between large-area DSSC modules and small-area cells in terms of preparative conditions and requirements. Hence, the effect of TiCl_4(aq)_ treatment on large-area DSSC modules needs to be discussed again.

In the process of enlarging the DSSC module, several conditions, such as the uniformity of the electrode, dye adsorption conditions, and module packaging methods, should be taken into consideration where the above-mentioned conditions are dissimilar to a cell with a small active area. When increasing the module area, the photocurrent in a large module could be hundreds of times of higher than in a small size cell. The most important issue in enlarging the DSSC module is how to efficiently collect the electron produced by the cell for the external circuit. Using metal wire with a transparent conductive coating (TCO) is one of the designs used to improve electron collection efficiency. However, several factors need to be considered in this aspect. First, the protective layer should be capped on the metal wire to avoid corrosion by electrolytes. Second, it is necessary to further consider the thermal expansion coefficient between the metal wire, the FTO transparent conductive substrate, and the protective layer during the TiO_2_ annealing process to achieve the best adhesion and coverage protection. Finally, the capping protective layer will affect the active area of the module, so that the aspect ratio and pitch of metal wire should be optimized to achieve the best module efficiency.

The other critical issue is the pattern design of the enlarged DSSC module. The goal of pattern design is to maximize the area of the working electrode, the so-called active area, to achieve the maximum power output. However, the metal grid and the gap between the metal fingers and the active area may reduce the active area, decreasing the power output of the device.

In this study, we propose to fabricate the highly efficient and stable DSSC module on the systematic exploration of optimized conditions for pre-treatment, post-treatment, and dye adsorption on large-area electrodes, and the design relevance on the performance of the enlarged DSSC module. On the other hand, a grid-type DSSC module is used as the basis of the metal wire design, where the photoanode is focused on. The fabrication of DSSC modules differs from that of single cells primarily due to the electrical connections among neighboring cells. Screen printing is usually employed as a coating method for the photoanodes of DSSC modules as it permits facile and controlled deposition in a few to 20 μm thickness range. This is a commercially available printing method and can be used for printing on both glass and plastic substrates. In addition, this technique offers compactness and good adhesion of TiO_2_ with the substrates, which otherwise often peels off from the surface in the annealing process. 

## 2. Materials and Methods

### 2.1. Chemicals and Reagents

Titanium tetrachloride (TiCl_4_), acetonitrile (AN), 3-methoxypropionitrile (MPN), 4-tert-butyl-pyridine (TBP), tert-butanol, iodine 99.8% (I_2_), chloroplatinic acid (H_2_PtCl_6_·6H_2_O), 1-propyl-2,3-dimethyl-imidazolium iodide (PMII), lithium perchlorate (LiClO_4_) and anhydrous lithium iodide (LiI) were used as received from Merck (Darmstadt, Germany) without further purification. Ethyl cellulose and α-terpineol were bought from Fluka (Buchs, Switzerland). Fluorine-doped tin oxide (FTO) glass with surface resistivity of 7 Ω cm^−2^ was purchased from Ruilong Optoelectronics Co.,Ltd (Miaoli Taiwan). The ruthenium polypyridyl photosensitizer Z907 was obtained from Everlight Chemical Industrial Co. (Taipei, Taiwan).

### 2.2. Fabrication of the DSSC Module

**Figure 1:**
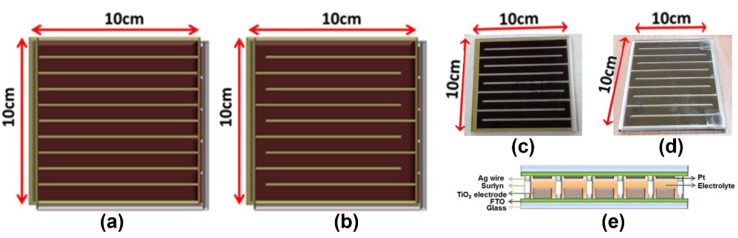
The sketch of DSSC module structure of (**a**) column-shaped (C-DSSC) module and (**b**) S-shaped SDDC (S-DSSC) module. The photo of (**c**) front and (**d**) rear side of 10 cm × 10 cm S-DSSC. (**e**) The cross-section sketch of DSSC module.

In this work, we compared two designs of grid-type DSSC modules: column-shaped (C-DSSC) modules and S-shaped SDDC (S-DSSC) modules. With the enlargement of the area of the DSSC module, the failure rate of electrolyte injection and the difficulty of packaging were enhanced because of an increase in the number of electrolyte injection holes for the strip electrode structure module (C-DSSC module). The other design of the S-shaped DSSC (S-DSSC) module was developed, also used by Fraunhofer Institute for Solar Energy Systems ISE in Germany [14], to enlarge the module area from a small-size module to a super-large module. By using this design, the number of electrolyte injection holes was decreased to only 2 and a higher electrode area per unit projection area was achieved. Figure 1a,b shows the schematic diagram of C-DSSC and S-DSSC module and the actual module photo of front and rear sides is shown in (c) and (d). Although S-DSSC design had such advantages, the distance between two electrolyte injection holes enhanced as the module area increased. As a result, the length of the electrolyte space between the two electrodes increased to boost the pressure difference in the module, which increased the difficulty of a fully electrolyte filling and air bubbles, where various sizes also accumulated between the two electrodes to block the electrochemical reaction in the module. In order to solve this problem, we used the cyclic injection method by using a peristaltic pumping to achieve the best packing condition for injecting the electrolyte (Figure S1); the module and electrolyte temperature were also controlled. The electrolyte pushed into module regularly could penetrate into the porous TiO_2_ electrodes and fill the mesopores in it. Finally, the two injection holes were sealed. To obtain the best filling condition, some factors such as electrolyte viscosity, volumetric expansion coefficient of solvent and the nano structure of TiO_2_ should be optimized at the same time.

In the basic design rules of the DSSC module shown in Figure 1e, the width and the thickness of Ag wire was 1 mm and 12 μm, respectively, whereas the width of the TiO_2_ working electrode (WE) and the Pt counter electrode (CE) was 6 mm. The WE were sandwiched together with CE separated by 60 µm thick hot-melt spacers (Surlyn, Dupont).

After cleaning, FTO glass was pre-treated by chemical bath deposition in titanium tetrachloride (TiCl_4_) diluted solution. The pre-treatment was processed at 70 °C for 30 min with 40 mM of TiCl_4(aq)_. The screen-printable TiO_2_ paste was prepared by thoroughly mixing 4.23 g TiO_2_ nanoparticles, 55 mL ethyl cellulose (EC), and 45 mL terpineol. The fluorine-doped tin oxide (FTO) substrates of working and pre-drilled counter electrodes (CEs) were cleaned using trichloroethylene (TCE), acetone (AC) and ethanol. Bi-layer TiO_2_ films with a selected TiO_2_/Ag grid pattern were sequentially screen-printed on FTO glass by using TiO_2_ paste as the active layer. A commercial TiO_2_ paste (JGC C&C PST400C, Kanagawa, Japan) was used as the light scattering layer with a commercial silver paste (EPI Technology, New Taipei City, Taiwan) for the current collection finger. The photo–anode was treated with different pre-treatment and post-treatment procedures involving TiCl_4(aq)_ at 70 °C for 30 min, and the thickness of TiO_2_ electrode was maintained to ca. 18 μm (14 μm + 4 μm) by controlling the cycles of the screen-printing process. A heat treatment of 120 °C after each coating cycle was helpful to stabilize each layer. Furthermore, the electrodes were heated at 500 °C for 30 min with atmospheric control and cooled at room temperature.

The post-treatment process was performed to improve the interconnection of TiO_2_ nanoparticles in the electrode. The annealed TiO_2_ electrode was dipped into 10 mM TiCl_4(aq)_ at 10 °C for 30 min followed by annealing at 500 °C for 30 min. The electrodes were then etched for series connections. A UV-treated paste screen-printed on Ag grid shape was cured by UV light for 1 min. The purpose of this coating was to protect the Ag grid line from possible damage in later processes such as dye soaking and electrolyte injection.

Then, the post-treated TiO_2_ electrode was immersed in a solution of 0.3 mM Z907 sensitizer solution in a mixture of AN and tert-butyl alcohol (*v:v* = 1:1) and kept at 50 °C for 12 h. The Z907-sensitized TiO_2_ electrode was then rinsed with AN to remove the remaining dye. A monolayer of catalytic platinum steered (500 °C, 30 min) after screen-printed by H_2_PtCl_6_ paste that consisted of EC and terpineol on pre-drilled FTO glass, served as the counter electrode (CE) of the DSSC. The morphology of Pt counter electrode is shown in Figure S2. The screen printing patterns of TiO_2_ WE and Pt WE are the same for cost-effective design [19]. The electrolyte used for measuring performance of the DSSC consisted of 0.8 M PMII, 0.04 M I_2_ and 0.5 M TBP in AN. Low-volatility electrolyte with a composition of 0.8 M PMII, 0.1 M I_2_, and 0.5 M TBP in MPN was used to test the reliability of the DSSC module. The dye-sensitized TiO_2_ electrode and counter electrode were separated by screen-printed UV adhesive in which it was used to fix the diffusion distance of the redox couple in electrolytes. The electrolyte was injected by peristaltic pumping method (Figure S1) to fully penetrate into the porous TiO_2_ electrodes and the module; electrolyte temperature was also controlled at the same time. Finally, the injection holes were sealed.

### 2.3. Characterization and Analysis

The conversion efficiency of DSSC module was measured by using a xenon lamp (Yamashita Denso, YSS-180S, Tokyo, Japan) with a light intensity of 100 mW cm^−2^ (AM1.5). The evolution of the electron transport mechanism in the module was investigated by using electrochemical impedance spectroscopy (EIS). The data of cyclic voltammetry (CV) and EIS were tested by using an electrochemical analyzer (Autolab, PGSTAT30, Utrecht, Netherlands). Impedance measurements were carried out by applying a DC bias at an open circuit voltage (V_OC_) and an AC voltage with an amplitude of 10 mV in a frequency range from 10^−2^ Hz–10^5^ Hz under light intensity of 100 mW cm^−2^ (AM1.5). For the voltammetric study, the measurement was carried out in 0.1 M LiI, 0.01 M I_2_ and 0.1 M LiClO_4_ in acetonitrile, FTO glass with or without treatment, platinum foil and Ag/Ag+ served as working, counter and reference electrodes, respectively. The reliability test of light soaking was carried under continuous visible light irradiation (100 mW cm^−2^) with the photovoltaic testing equipment (KD-SACL-0404, KING DESIGN, New Taipei City, Taiwan). The morphologies of the electrode surface were characterized by a Scanning Electron Microscope (SEM, JEOL JSM-7000, Tokyo, Japan). The transmittance measurement was performed by UV-VIS spectrometer (V-670, Jasco, Easton, MD, USA).

## 3. Results and Discussion

### 3.1. Effect of TiCl_4(aq)_ Involving Pre-Treatment and Post-Treatment

**Figure 2:**
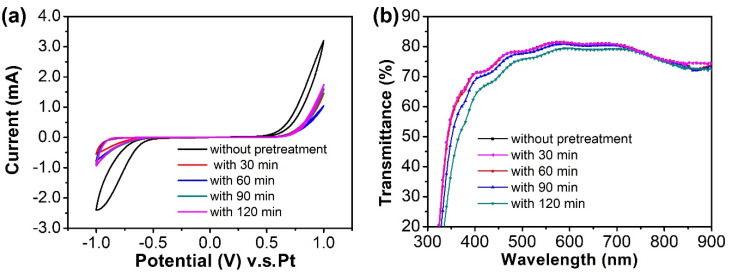
FTO glass pre-treatment by TiCl_4(aq)_ for different times. (**a**) CV analysis and (**b**) Transmittance measurement.

First, the FTO glass was immersed into TiCl_4(aq)_ solution at 70 °C and, as a result, the TiO_2_ dense layer of several nanometers grew as a pre-treatment process before printing the TiO_2_ electrode. After pre-treatment, the connection between the porous TiO_2_ electrode and FTO glass with pre-treatment was also improved to reduce the series resistance caused by the interface. At the same time, the TiO_2_ dense layer also inhibited the contact between FTO and I^-^/I_3_^-^ ions in the electrolyte to reduce the direct recombination reaction of electrons from FTO glass and I_3_^-^ ion. From the CV-scan analysis shown in Figure 2a, the higher current of FTO glass without pre-treatment indicates a better electrocatalytic activity for the reduction of I_3_^-^ ions in the electrolyte solution with the electron on FTO glass. For the same reason, at the same voltage, the significantly lower reaction current measured in the sample with pre-treatment proves that the dense TiO_2_ layer between the FTO glass and porous TiO_2_ film could effectively inhibit the recombination between electrons on FTO and I_3_^-^ ions in the electrolyte solution. Yet, it was observed that a longer pre-treatment time resulted in a higher reaction current. This was explained by the rough surface of thick dense TiO_2_ layer. Although the modified TiO_2_ dense layer on conductive FTO glass could reduce the electron recombination reaction on FTO, the rougher and thicker TiO_2_ dense layer could also increase the interface resistance to affect the barrier effect of the TiO_2_. On the other hand, the thicker TiO_2_ dense layer also reduces the light transmittance of substrates (shown in Figure 2b) to decrease the module performance. As shown in in Table 1, the short-circuit current density was inversely proportional to the pre-treatment time which was affected by the transmittance of FTO glass. Yet, it was obvious that the PV parameters such as J_SC_, V_OC_ and FF were all improved by TiCl_4_ pre-treatment. After TiCl_4_ pretreatment, a dense TiO_2_ layer was formed on FTO glass to avoid carrier recombination between I_3_^−^ and FTO effectively. Additionally, the dense TiO_2_ layer also played a role in improving the network bonding strength between TiO_2_ nanoporous film and FTO substrates. Small particles of TiO_2_ produced by TiCl_4_ pretreatment could be connected with the dead-end TiO_2_ nanoporous film and therefore enhance electron transport. 

**Figure 3:**
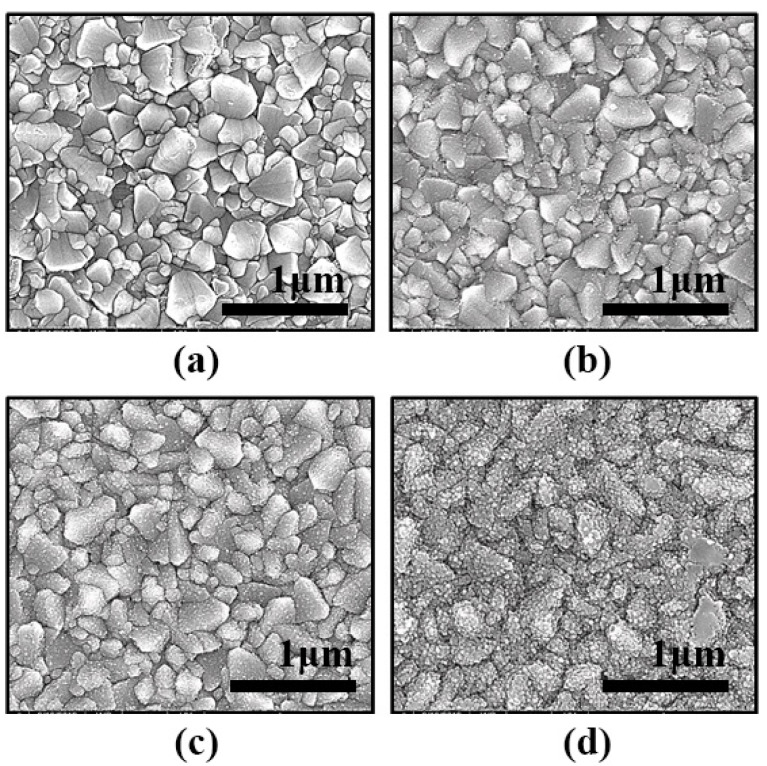
SEM images of FTO glass pre-treatment by different concentrations of TiCl_4(aq)_: (**a**) 0 mM, (**b**) 40 mM, (**c**) 200 m M and (**d**) 400 mM.

Furthermore, the morphology of the TiO_2_ dense layer formed on FTO conductive glass was affected by the concentration of TiCl_4(aq)_. At a high concentration of TiCl_4(aq)_, violent reaction affected the structure stack of the TiO_2_ layer on FTO glass, whereas a flatter TiO_2_ dense layer could be smoothly grown in low concentration TiCl_4(aq)_ solution. This phenomenon was also observed in SEM images of Figure 3. The proper TiO_2_ thickness of pre-treatment could meet the requirements for blocking the recombination on the surface of FTO glass, and the high thickness of TiO_2_ film reduces the amount of light incidence and increased resistance. The best efficiency is achieved under the pre-treatment condition with 0.04 M TiCl_4(aq)_ and 70 °C for 30 min reaction time.

**Figure 4:**
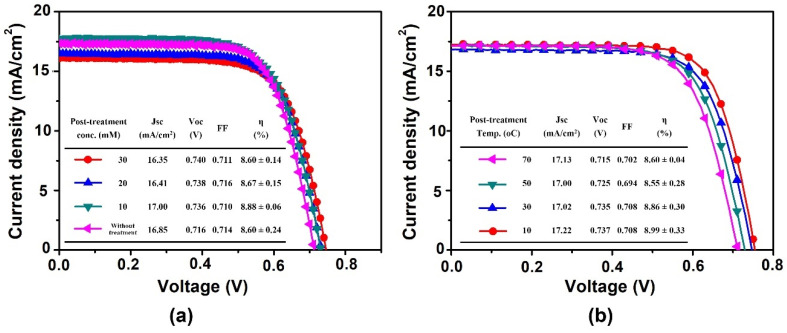
J-V curve of 5 cm × 5 cm DSSC module with TiCl_4(aq)_ post-treatment at (**a**) different concentrations and (**b**) different temperatures.

**Figure 5:**
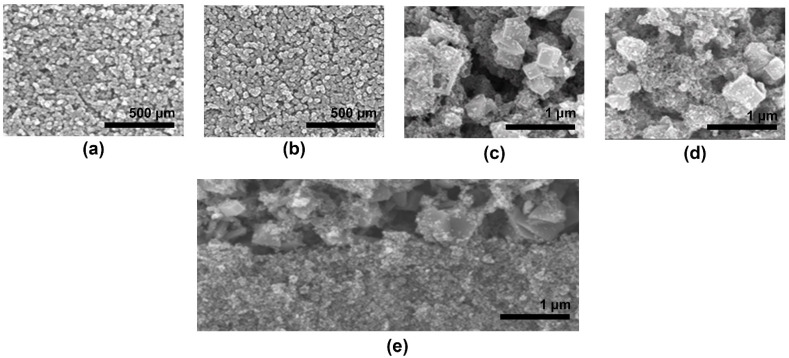
SEM images of the top view of TiO_2_ electrode without (**a**,**c**) and with (**b**,**d**) TiCl_4(aq)_ post-treatment. (**e**) The cross-section of meso-TiO_2_ electrode on FTO glass.

Figure 4a summarizes the photovoltaic properties of the TiO_2_ film with the various TiCl_4_-concentration post-treatment. The suitable post-treatment concentration of TiCl_4_ could improve both J_SC_ and V_OC_ obviously. The V_OC_ increased with a high TiCl_4_ concentration due to the improvement of the connection between the TiO_2_ nanoparticles. On the other hand, J_SC_ improvement was dependent on the concentration of TiCl_4_ because of the presence of a considerable amount of dye soaking on changing the pore size of the porous TiO_2_ electrode. The DSSC module treated with a 10 mM TiCl_4(aq)_ post-treatment concentration exhibited the largest power conversion efficiency (avg. = 8.88%), resulting primarily from the trade-off between the J_SC_ and V_OC_. In SEM image of Figure 5, the larger size TiO_2_ nano particles on TiO_2_ electrodes were produced in a high concentration TiCl_4(aq)_ solution due to a violent reaction. This phenomenon is just like the pre-treatment, as mentioned in the previous discussion. Through the post-treatment, the connectivity of the modified TiO_2_ electrode could be improved to reduce the possibility of electron-hole recombination leading to an increase in the V_OC_. 

The nanopore size of the mesoporous TiO_2_ electrode post-treated at a high concentration TiCl_4(aq)_ is too small to diffuse into the ions of the electrolyte. The I_3_- cannot quickly diffuse into the reducde electrode, Pt, to increase the probability of the electron recombination and to reduce the V_OC_ of the device.

In addition to the investigations associated with the use of different concentrations of TiCl_4(aq)_ previously, the reaction temperature of the post-treatment was also an important variable for controlling the TiCl_4(aq)_ reaction. Through studying the reaction concentration, the reaction behavior of the post-treatment could be systematically realized. As shown in Figure 4b, TiCl_4_ reacts slowly at 10 °C and a uniform film was formed on the porous TiO_2_ electrode, which could successfully avoid the electron recombination reaction. While at 70 °C (vigorous TiCl_4_ reaction), more TiO_2_ thin film reacted on the TiO_2_ particles and the pore size was reduced, resulting in a diffusion of ions in the porous TiO_2_ electrode with more difficulty. This resulted in a decrease in the V_OC_ of the DSSC module as the post-treatment temperature increased.

### 3.2. Effect of Dye Soaking Condition

**Figure 6:**
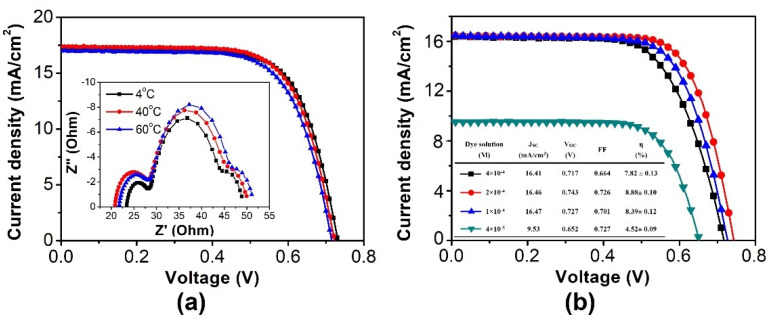
(**a**) The J-V curve with different dye soaking temperatures and the insert plot (Nyquist plot of EIS analysis). (**b**) The J-V curve of DSSC module with TiO_2_ electrode dye-soaking in different dye concentrations with detailed performance data.

Because the dyes are the main source of photocurrent after visible light absorption, it is important to understand how to transport photocurrent from the TiO_2_ electrode to the external circuit effectively to impact the conversion efficiency of the DSSC module. By controlling the dye sensitization process, the ideal monolayer dye can be formed in the TiO_2_ electrode to reduce the light energy waste caused by dye aggregation and to increase the V_OC_ and FF of the DSSC module. In this study, the Z907 dye sensitizer has a hydrophobic side chain group to reduce the dye aggregation effect. During the dye sensitization, the side chain group can not only avoid the dye aggregation but also, decrease the possibility of the recombination reaction between the electrons, oxidizing dye molecules to improve the module stability. So, the monolayer Z907 dye molecules can be sensitized on the TiO_2_ electrode by controlling the sensitized temperature and the concentration of dye solution. Under high-temperature conditions, dye molecules have a higher kinetic energy to not only accelerate the dye sensitization process but also cause the dye aggregation situation. On the contrary, the dye is sensitized to the monolayer regularly to decrease the possibility of the dye-aggregation phenomenon under low-temperature conditions. This behavior can also reduce the recombination reaction between electrons and oxidized dyes. In Figure 6a, the V_OC_ and FF of the module increases with the decrease in the dye soaking temperature. This is explained by the Nyquist plot inserted in Figure 6a. In the second semicircle of the Nyquist plot, the charge transfer resistance at the TiO_2_ electrode/electrolyte interface (R_ct2_) is fitted. It is demonstrated that the lower process temperature is 60 °C to 40 °C to 4 °C and the lower R_ct2_ is 21.4 ohm to 19.0 ohm to 16.5 ohm. This phenomenon proves that lowering the temperature of the sensitization process can not only help the dye molecules to become neatly arranged on the TiO_2_ electrode but also reduces dye aggregation and the electron recombination reaction.

**Figure 7:**
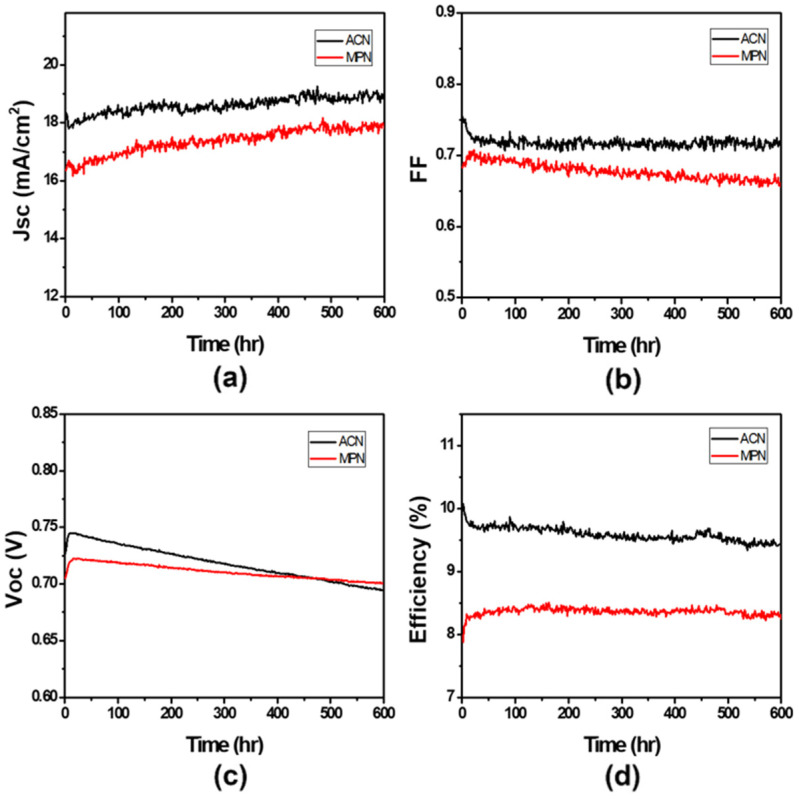
Time evolution of the (**a**) J_SC_, (**b**) FF, (**c**) V_OC_ and (**d**) efficiency of a 5 cm × 5 cm C-DSSC module under continuous one-sun light soaking at 60 °C and 60 RH%.

On the other hand, the usage of dye is related to the module cost and the uniformity condition of dye on TiO_2_ electrode. So, the concentration of the dye solution needs to be discussed to clarify the module performance affected by the bonding behavior between the dye and TiO_2_ electrode. In a series of experiments, we observed that the module current is little bit lower by decreasing the concentration of dye solution. On the other hand, the V_OC_ and FF of the module are clearly improved. Figure 6b shows the non-uniform dye soaking on the TiO_2_ electrode because the dye concentration of 4 × 10^−5^ M is too low. Clearly, part of the TiO_2_ electrode is exposed without dye sensitization to decrease the J_SC_ and V_OC_ of module. The dyes are especially dispersed homogeneously in solution at a proper dye concentration of 2 × 10^−4^ M. The dye molecule has plenty of time and space to form the monolayer of Z907 dye on the TiO_2_ electrode. When dye sensitizing was conducted properly, the V_OC_ and FF of the module improved, and the efficiency increased to 8.88%. In this study, the module efficiency of 8.7% was achieved for a 5 cm × 5 cm grid-type C-DSSC module that was independently verified by the Research Center for New Generation PhotoVoltaics (RCNPV) of National Central University, Taiwan (Figure S3). Moreover, 5 cm × 5 cm C-DSSC module with AN- and MPN-based electrolytes is also under light soaking for stability test by using one sun simulated light source at 60℃ and 60 RH% condition. From Figure 7, we demonstrated that the initial module efficiency of a C-DSSC module with an AN-based electrolyte was achieved at approximately 10% and the outstanding efficiency after light soaking was kept >9.5%. The reason for long-term stability efficiency drop was mainly explained by the leakage of electrolytes from the DSSC device due to its low boiling point. On the other hand, a good solvent should have a high dielectric constant for the sufficient dissolution of the ionic salts, and its acceptable melting point should range from −20 °C to 100 °C for survival in extreme outdoor conditions. In addition to AN, other nitrile solvents with a high boiling point and lower toxicity, such as MPN, have such properties.

Widely used as alternatives to traditional electrolytes. In this study, the initial efficiency of a C-DSSC module with an MPN-based electrolyte achieved around 8% efficiency. After >20 h of light soaking, the module efficiency greatly increased and remained at 8.5% with improved J_SC_, V_OC_ and FF, due to the complete electrolyte penetration into the porous TiO_2_ electrode. The obvious trend in the difference of the efficiency, initially and after >20 h light soaking under AN- and MPN-based electrolyte systems, mainly stemmed from the solvent viscosity. A higher solvent viscosity caused a slower electrolyte penetration and equilibrium speed of the electrode potential. Hence, after making the C-DSSC module and subjecting it to light soaking for >20 h, the stable photovoltaic performance can be measured. Under the better protection from Ag wire of a module which has good-sealed tech, the efficiency of the C-DSSC module with both AN- and MPN-based electrolytes can be kept at a stable performance under continuous 600 h light soaking at 60 °C and 60 RH% condition.

### 3.3. Size effect of DSSC Modules

**Figure 8:**
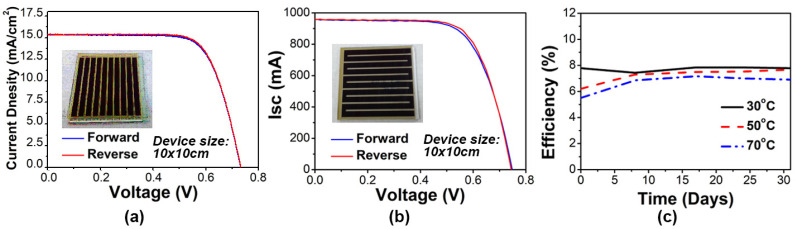
Stability photovoltaic performance of a 10 cm × 10 cm (**a**) C-DSSC and (**b**) S-DSSC module. (Insert: actual module photo picture.) (**c**) The efficiency trend of dye-sensitized solar modules at different temperatures of electrolyte injection.

After the optimization of 5 cm × 5 cm C-DSSC modules, module area is further enlarged to test the process parameters, the reliability and the sealing method during the enlargement of C-DSSC module. Figure 8a shows the J-V curve of the 10 cm × 10 cm C-DSSC module and its genuine images. Keeping the same aspect ratio of Ag wire, the electrode and the pitch between the Ag wires to the electrode screen-printed onto the FTO substrate, the module is enlarged to 10 cm × 10 cm by increasing the length and number of the Ag wire electrodes. The active area of the module is increased to 48.70 cm^2^. The hysteresis effect of the J-V curve by forward and backward scanning is not observed even when the module is enlarged to a 10 cm × 10 cm size and a module efficiency of 8.1% is achieved. This further enlarges the module area to a 10 cm × 15 cm size with an active area of 83.1 cm^2^. In Table 2, the efficiency of a 10 cm × 15 cm C-DSSC module reaches an outstanding value of 8.06% with 1240.64 mA of I_SC_, 0.722 V of V_OC_, 669.76 mW of P_MAX_ and 0.748 of FF. Although the efficiency of an S-DSSC-100 module is only 7.9% (slightly lower than that of C-DSSC), an S-DSSC-100 module (64.85 cm^2^) can have a larger active area than a C-DSSC-100 module (48.70 cm^2^) on the same 10 cm × 10 cm FTO glass substrate. The increased area derives from the turning point of the S-shaped channel. It is believed that the larger WE area may lead to a greater power output of 512.32 mW in an S-DSSC-100 module, compared to the 395.17 mW in a C-DSSC-100 module under the same device size (10 cm × 10 cm), for higher photo current (I_SC_) and power generation (P_MAX_). To enlarge the DSSC module, there are two possible directions to choose from: one is to lengthen the strip length, and the other is to increase the strip numbers and fix the strip width simultaneously. As per the results (Table S1), the Isc is proportional to the area of the working electrode (WE), but the strip length affects all of the PV parameters. The longer the strip length is, the lower the J_SC_, V_OC_, FF, and efficiency of the device; this is the implication for the traffic jam of electrons in the Ag wire (finger). To solve this problem, the pitch and cross-section of the Ag wire should be reconsidered and optimized. On the other hand, increasing the strip numbers seems to be an action independent of Voc, Jsc, FF and the efficiency of the device. The reason is that the bus bar is wide enough to collect current from the Ag wire (finger). Based on these results (Table S1), compared to the C-DSSC-100 (10 strips) and C-DSSC-150 (16 strips), the change of PV performance is independent from the increased width due to the same electron collection condition for each strip. Another key difference between the S-DSSC and the C-DSSC, is that the C-DSSC-100 has 10 strips (check the detail in the schematic diagram in Figure 1), and each strip has two holes, so there are 20 holes in total for the electrolyte injection. On the other hand, in S-DSSC-100, there is a hole at the beginning and at the end. There are only two holes in the S-DSSC design, regardless of the size of the device. The authors suggest that the possibility of electrolyte leakage in the C-DSSC-100 (20 holes) is higher than that in the S-DSSC-100 (two holes only) due to the sealing issue. Hence, the S-DSSC module is a recommended design for commercialization and mass production due to the advantage of easier packaging and electrolyte injection on enlarging the module and achieving a higher power generation per unit area. However, the complex pattern of the S-DSSC is a disadvantage, demonstrating that the S-DSSC cannot inject the electrolyte perfectly. This disadvantage should be considered until the peristaltic pump is used.

As seen from Figure 8c, injecting the electrolyte at higher temperatures, for example 50 °C and 70 °C, reduces the viscosity of the electrolyte, which helps the electrolyte to penetrate easily into the mesoporous TiO_2_ electrodes. When the temperature of the S-DSSC module is lowered from 50 °C and 70 °C to room temperature (~25 °C) after the filling procedure, many small bubbles are produced in the electrodes due to the shrinkage of the electrolyte volume (Figure S4) from the volumetric expansion coefficient of the solvent. The inefficient electrolyte filling results in a slight decrease in the initial efficiency of the S-DSSC module with a higher preparation temperature. It takes about 150 h to reach equilibrium and increase to a stable efficiency due to electrolyte re-permeation. Therefore, the injecting temperature should be controlled at 30 °C until the injection process is completed to achieve the best efficiency and stability of the S-DSSC module.

## 4. Conclusions

In this study, we presented the procedure for manufacturing a high-performance DSSC module step by step from the pre- and post-treatment of the TiO_2_ electrode, the dye absorption condition and the electrolyte filling condition. All these process conditions may affect the performance of the DSSC module. The performance of the dye-sensitized condition of the TiO_2_ photoanode in a 5 cm × 5 cm C-DSSC module was investigated. Before the screen printing of the TiO_2_ photoanode, a compact TiO_2_ underlayer was applied for a 30 min pretreatment of the FTO substrate sprayed from a 40 mM TiCl_4_ solution at 70 °C. The recombination and dark current was effectively suppressed by the TiO_2_ underlayer to improve the Jsc and Voc of the module. After the post-treatment by using 10 mM TiCl_4_ solution at 10 °C, the post-treatment effectively modified the connectivity between porous titanium dioxide electrode particles. Because the electron on the electrode could be collected at the external circuit to reduce the recombination reaction by the post-treatment process, the module Voc and the efficiency could be increased. By using a 2 × 10^−4^ M Z-907 dye solution at 4 °C, the sensitization behavior tended to become slow to achieve the ideal Z-907 monolayer on the TiO_2_ photoanode. This process can also improve the Voc and FF of the module to increase its efficiency.

In a large DSSC module study, the design of an S-shape DSSC module was also demonstrated with several advantages, such as less possibility of electrolyte leakage and a large active area to achieve a high-performance DSSC module. The efficiency of 8.88% for a 5 cm × 5 cm C-DSSC module was achieved, in addition to the conversion efficiency of 10 cm ×10 cm and 10 cm ×15 cm modules, which could be kept at the high performances of 8.14% and 8.06%, respectively. Under continuous light soaking for 600 h, the sealed modules maintained excellent and stable performances with almost unchanged module efficiency. Compared to the C-DSSC module, the structure of the S-DSSC module can reduce the number of electrolyte injection holes to decrease the possibility of electrolyte leakage and improve the enhancement of the effective power generation area. On the other hand, the distance of the Ag electrode, required to collect electrons in the S-DSSC module, was longer than in the C-DSSC module; the FF of S-DSSC was slightly lower and its efficiency slightly decreased to 7.9%. Overall, the power of the S-DSSC-100 module was 512 mW, which was clearly higher than the 396 mW of the C-DSSC-100 module. Finally, the best module performance and stability of the S-DSSC module was achieved by controlling the electrolyte injection temperature at 30℃. The electrolyte filling issue of the S-DSSC module can be solved by the peristaltic pumping method. Overall, the design of S-DSSC module is better than the C-DSSC. It is believed that this design and its concept should be widely used in all DSSC modules.

## Figures and Tables

**Table 1:** Performance of devices at different TiCl_4(aq)_ pre-treatment time.

Pre-Treatment Time (min)	Jsc (mA/cm^2^)	Voc (V)	FF	η (%)
0	15.60	0.725	0.722	8.17 ± 0.06
30	16.89	0.732	0.718	8.86 ± 0.03
60	16.34	0.740	0.721	8.71 ± 0.27
90	15.92	0.735	0.719	8.41 ± 0.21

**Table 2:** PV parameters of various-sized grid-type DSSC modules (MPN based ELE.) under AM1.5 G illumination.

Module	Active Area (cm^2^)	Scan Direction	P_MAX_ (mW)	I_SC_ (mA)	J_SC_ (mA/cm^2^)	V_OC_ (V)	FF	Efficiency (%)
C-DSSC-100	48.67	Forward	395.17	745.57	15.32	0.733	0.723	8.12
C-DSSC-100		Reverse	396.15	745.58	15.32	0.733	0.725	8.14
C-DSSC-150	83.10	Forward	666.40	1240.66	14.93	0.720	0.746	8.02
C-DSSC-150		Reverse	669.76	1240.64	14.93	0.722	0.748	8.06
S-DSSC-100	64.85	Forward	512.32	958.46	14.78	0.744	0.719	7.90
S-DSSC-100		Reverse	499.99	958.10	14.77	0.747	0.699	7.71

## Data Availability

The data supporting the findings of this study are available within the article or its supplementary materials.

## Supplementary Materials

The following are available online at https://www.mdpi.com/article/10.3390/nano11082125/s1. Figure S1: Schematic illustration of the peristaltic pump to continuously inject electrolytes into the DSSC module; Figure S2. The SEM image of Pt particles on FTO glass; Figure S3: Efficiency evaluation of dye-sensitized solar module (module size = 5 cm × 5 cm, black metal mask active area = 6.658 cm^2^) by RCNPV in NCU, Taiwan; Figure S4: Photo of S-DSSC modules with and without bubbles after cyclic injection electrolyte. Table S1: The PV performance of devices with different enlarged direction; Detail process of Pt counter electrode.
