# Supplementary material for: Controlled Photoanode Properties for Large-Area Efficient and Stable Dye-Sensitized Photovoltaic Modules
Source: Nanomaterials (Basel). 2021 Aug 20;11(8):2125. doi: 10.3390/nano11082125 (PMC8398926; doi:10.3390/nano11082125)
Supplement: Supplementary file 1 [file nanomaterials-11-02125-s001.zip › nanomaterials-1340514-supplementary.pdf]

*nanomaterials*

*Article*

# Control Photoanode Properties for Large Area Efficient and Stable Dye-sensitized Photovoltaic Modules

**Wei-Hao Chiu [1,†], Kun-Mu Lee[1,2,3,*,†], Vembu Suryanarayanan [4], Jen-Fu Hsu [3,5,*] and Ming-Chung Wu [1,2,3,*]**

1 Center of Green Technology, Chang Gung University, Taoyuan, 33302, Taiwan; weihao.chiu@gmail.com
2 Department of Chemical and Materials Engineering, Chang Gung University, Taoyuan, 33302 Taiwan
3 Division of Neonatology, Department of Pediatrics, Chang Gung Memorial Hospital, Linkou, 33305, Taiwan
4 Electroorganic and Materials Electrochemistry Division, CSIR-Central Electrochemical Research Institute, Karaikudi 630003, India; vidhyasur@yahoo.co.in
5 School of Medicine, College of Medicine, Chang Gung University, Taoyuan 33302, Taiwan
* Correspondence: kmlee@mail.cgu.edu.tw (K.-M.L.); hsujanfu@cgmh.org.tw (J.-F.H.); mingchungwu@mail.cgu.edu.tw (M.C. Wu); Tel.: +886-3-2118800#3891 (K.-M.L.); Tel.: +886-3-3281200 (J.-F.H.); Tel.: +886-3-2118800#3545 (M.-C.W.)
† These authors contributed equally to this work.

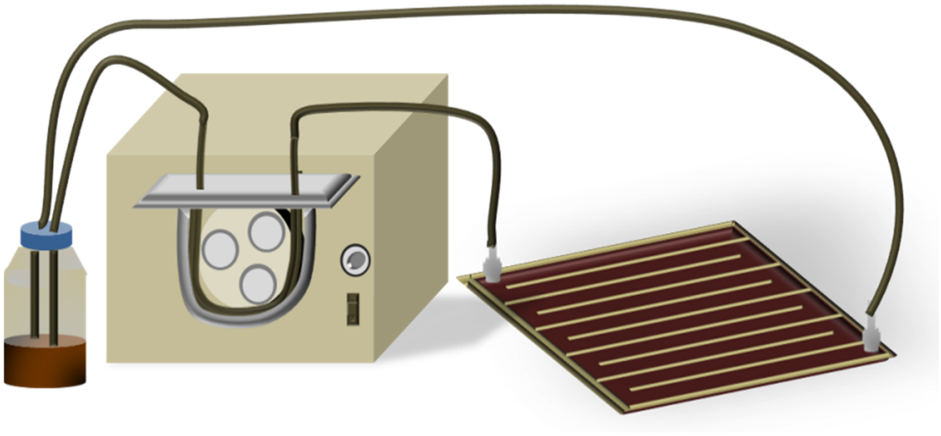

**Figure S1**. Schematic illustration of the peristaltic pump to continuously inject electrolyte into the DSSC module.

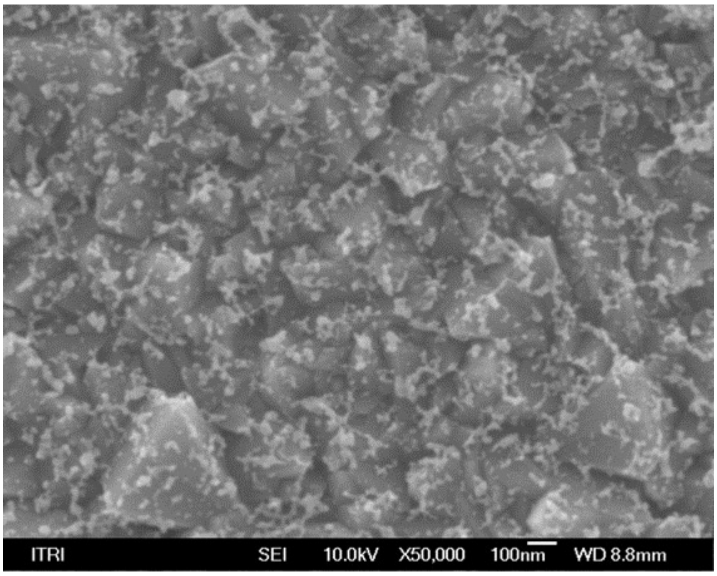


**Figure S2.** The SEM image of Pt particles on FTO glass.

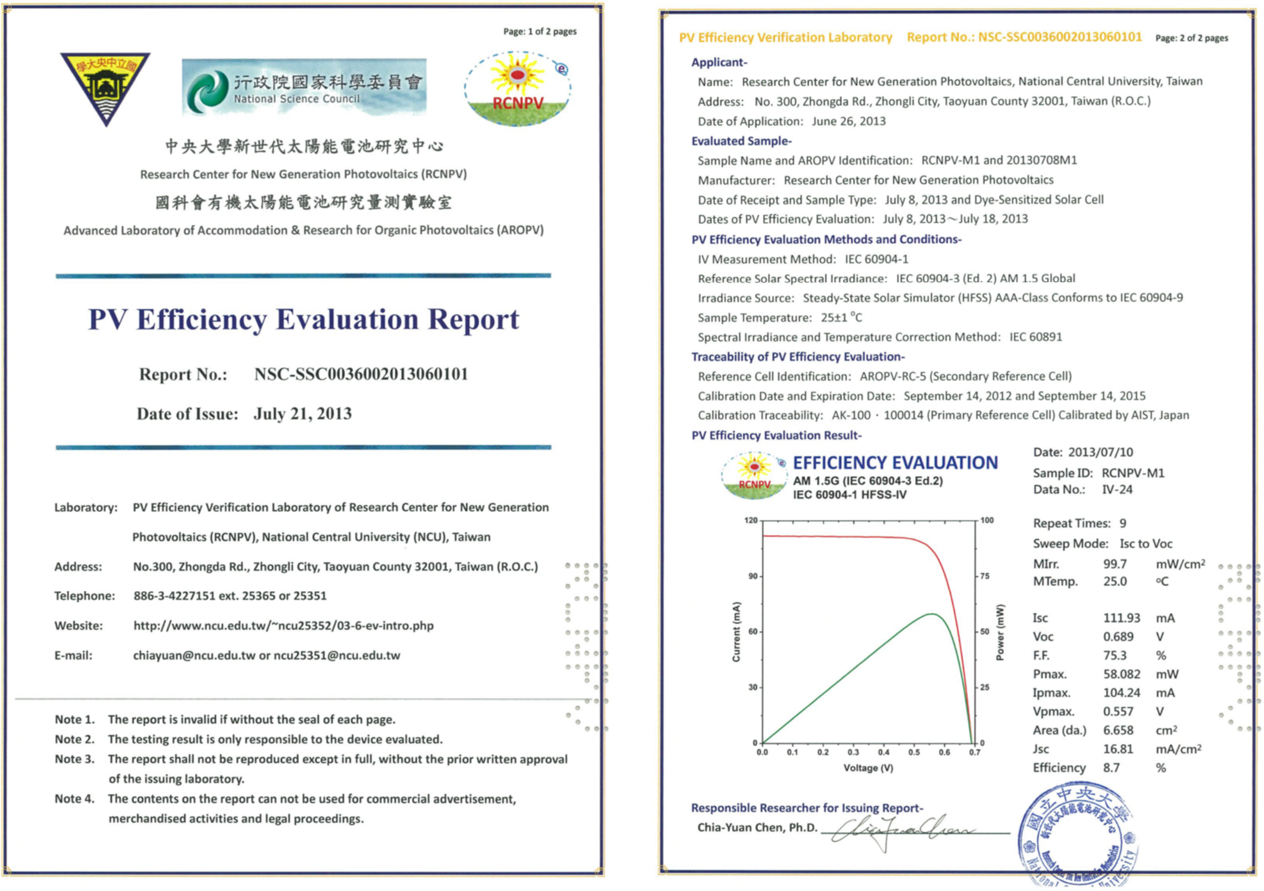
Page: 1 of 2 pages

行政院國家科學委員會
National Science Council

RCNPV

中央大學新世代太陽能電池研究中心

Research Center for New Generation Photovoltaics (RCNPV)

國科會有機太陽能電池研究量測實驗室

Advanced Laboratory of Accommodation & Research for Organic Photovoltaics (AROPV)

**PV Efficiency Evaluation Report**

Report No.: NSC-SSC0036002013060101

Date of Issue: July 21, 2013

Laboratory: PV Efficiency Verification Laboratory of Research Center for New Generation Photovoltaics (RCNPV), National Central University (NCU), Taiwan

Address: No.300, Zhongda Rd., Zhongli City, Taoyuan County 32001, Taiwan (R.O.C.)

Telephone: 886-3-4227151 ext. 25365 or 25351

Website: http://www.ncu.edu.tw/~ncu25352/03-6-ev-intro.php

E-mail: chiayuan@ncu.edu.tw or ncu25351@ncu.edu.tw

Note 1. The report is invalid if without the seal of each page.

Note 2. The testing result is only responsible to the device evaluated.

Note 3. The report shall not be reproduced except in full, without the prior written approval of the issuing laboratory.

Note 4. The contents on the report can not be used for commercial advertisement, merchandised activities and legal proceedings.

PV Efficiency Verification Laboratory Report No.: NSC-SSC0036002013060101 Page: 2 of 2 pages

Applicant-

Name: Research Center for New Generation Photovoltaics, National Central University, Taiwan

Address: No. 300, Zhongda Rd., Zhongli City, Taoyuan County 32001, Taiwan (R.O.C.)

Date of Application: June 26, 2013

Evaluated Sample-

Sample Name and AROPV Identification: RCNPV-M1 and 20130708M1

Manufacturer: Research Center for New Generation Photovoltaics

Date of Receipt and Sample Type: July 8, 2013 and Dye-Sensitized Solar Cell

Dates of PV Efficiency Evaluation: July 8, 2013～July 18, 2013

PV Efficiency Evaluation Methods and Conditions-

IV Measurement Method: IEC 60904-1

Reference Solar Spectral Irradiance: IEC 60904-3 (Ed. 2) AM 1.5 Global

Irradiance Source: Steady-State Solar Simulator (HFSS) AAA-Class Conforms to IEC 60904-9

Sample Temperature: 25±1 °C

Spectral Irradiance and Temperature Correction Method: IEC 60891

Traceability of PV Efficiency Evaluation-

Reference Cell Identification: AROPV-RC-5 (Secondary Reference Cell)

Calibration Date and Expiration Date: September 14, 2012 and September 14, 2015

Calibration Traceability: AK-100 · 100014 (Primary Reference Cell) Calibrated by AIST, Japan

PV Efficiency Evaluation Result-

EFFICIENCY EVALUATION
AM 1.5G (IEC 60904-3 Ed.2)
IEC 60904-1 HFSS-IV



Date: 2013/07/10

Sample ID: RCNPV-M1

Data No.: IV-24

Repeat Times: 9

Sweep Mode: Isc to Voc

| | | |
|---|---|---|
| MIrr. | 99.7 | mW/cm² |
| MTemp. | 25.0 | ºC |
| Isc | 111.93 | mA |
| Voc | 0.689 | V |
| F.F. | 75.3 | % |
| Pmax. | 58.082 | mW |
| Ipmax. | 104.24 | mA |
| Vpmax. | 0.557 | V |
| Area (da.) | 6.658 | cm² |
| Jsc | 16.81 | mA/cm² |
| Efficiency | 8.7 | % |

Responsible Researcher for Issuing Report-

Chia-Yuan Chen, Ph.D.

**Figure S3.** Efficiency evaluation of dye-sensitized solar module (module size = 5cm × 5cm, black metal mask active area = 6.658 cm2) by RCNPV in NCU, Taiwan.

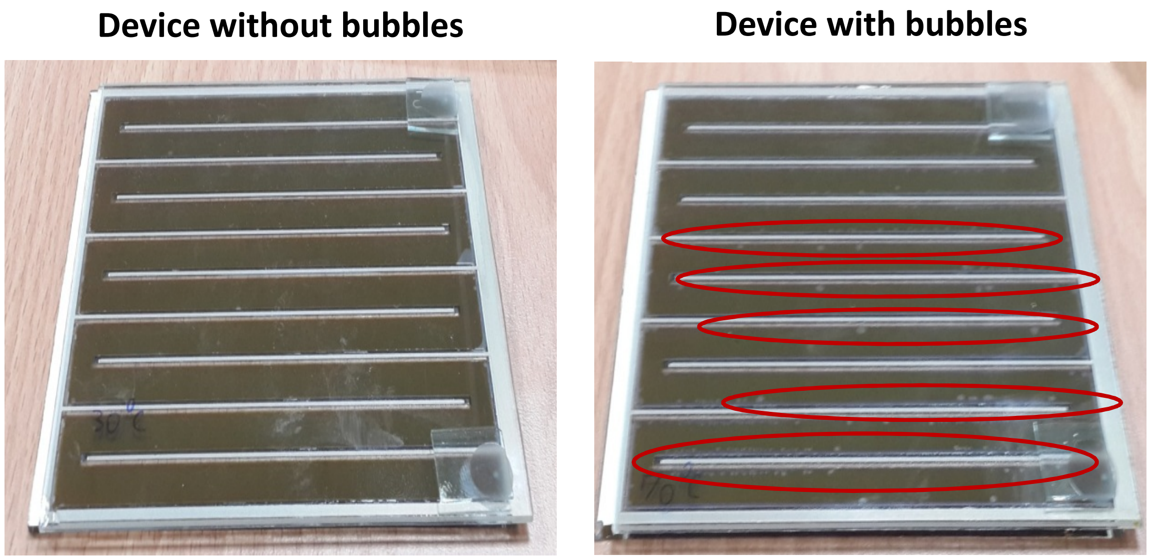


**Figure S4.** Photo of S-DSSC modules with (right) and without (left) bubbles after cyclic injection electrolyte.

**Table S1.** The PV performance of devices with different enlarged direction.

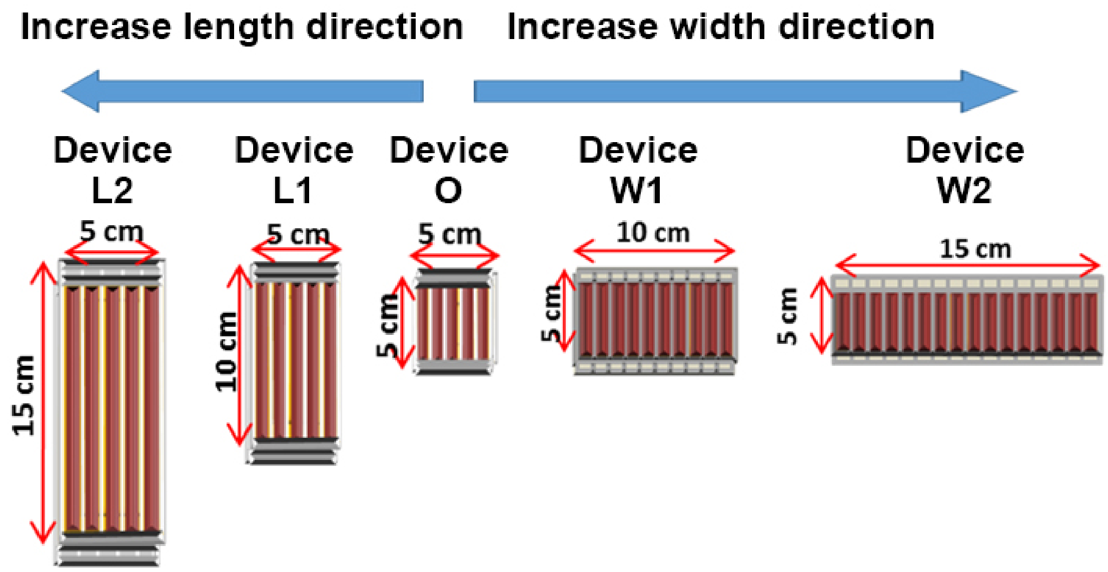


| Device | Length x width x numbers (cm x cm x numbers) | Isc (mA) | $J_{SC}$ (mA/cm$^2$) | $V_{OC}$ (V) | FF | Efficiency (%) |
|---|---|---|---|---|---|---|
| Device O | 3.6 x 0.6 x 5 | 165.71 | 15.34 | 0.747 | 0.701 | 8.03 |
| Device W1 | 3.6 x 0.6 x 10 | 341.48 | 14.98 | 0.710 | 0.728 | 7.74 |
| Device W2 | 3.6 x 0.6 x 16 | 604.54 | 15.10 | 0.729 | 0.721 | 7.94 |
| Device L1 | 8.9 x 0.6 x 5 | 392.92 | 14.73 | 0.734 | 0.700 | 7.58 |
| Device L2 | 13.9 x 0.6 x 5 | 618.59 | 14.94 | 0.722 | 0.687 | 7.42 |

**Detail process of Pt counter electrode**

First, polymer ethyl cellulose (EC) was dissolved in terpineol at a content ratio of 10.0 wt.% and stirred vigorously for 24 hours by an external rotating magnet. $H_2PtCl6$ is added into 10.0 wt.% EC/terpineol solution with concentration of $2{\times}10^{-2}$ M and uniformly dispersed by a rotating magnet. Second, the patterned Pt counter electrode is screen-printed on drilled FTO glass by using the prepared Pt paste. Finally, the Pt counter electrodes were heated at 500 ℃ for 30 minutes with atmospheric control and cooled at room temperature.
